# The directionality of the nuclear transport of the influenza A genome is driven by selective exposure of nuclear localization sequences on nucleoprotein

**DOI:** 10.1186/1743-422X-6-68

**Published:** 2009-06-02

**Authors:** Winco WH Wu, Nelly Panté

**Affiliations:** 1 Department of Zoology, University of British Columbia, 6270 University Boulevard, Vancouver, British Columbia, V6T 1Z4, Canada

## Abstract

**Background:**

Early in infection, the genome of the influenza A virus, consisting of eight complexes of RNA and proteins (termed viral ribonucleoproteins; vRNPs), enters the nucleus of infected cells for replication. Incoming vRNPs are imported into the nucleus of infected cells using at least two nuclear localization sequences on nucleoprotein (NP; NLS1 at the N terminus, and NLS2 in the middle of the protein). Progeny vRNP assembly occurs in the nucleus, and later in infection, these are exported from the nucleus to the cytoplasm. Nuclear-exported vRNPs are different from incoming vRNPs in that they are prevented from re-entering the nucleus. Why nuclear-exported vRNPs do not re-enter the nucleus is unknown.

**Results:**

To test our hypothesis that the exposure of NLSs on the vRNP regulates the directionality of the nuclear transport of the influenza vRNPs, we immunolabeled the two NLSs of NP (NLS1 and NLS2) and analyzed their surface accessibility in cells infected with the influenza A virus. We found that the NLS1 epitope on NP was exposed throughout the infected cells, but the NLS2 epitope on NP was only exposed in the nucleus of the infected cells. Addition of the nuclear export inhibitor leptomycin B further revealed that NLS1 is no longer exposed in cytoplasmic NP and vRNPs that have already undergone nuclear export. Similar immunolabeling studies in the presence of leptomycin B and with cells transfected with the cDNA of NP revealed that the NLS1 on NP is hidden in nuclear exported-NP.

**Conclusion:**

NLS1 mediates the nuclear import of newly-synthesized NP and incoming vRNPs. This NLS becomes hidden on nuclear-exported NP and nuclear-exported vRNPs. Thus the selective exposure of the NLS1 constitutes a critical mechanism to regulate the directionality of the nuclear transport of vRNPs during the influenza A viral life cycle.

## Background

The influenza A virus exploits the cellular nuclear transport machinery several times during infection (reviewed in [1]). Early in infection, the influenza A viral genome – consisting of eight complexes of RNA and proteins (ribonucleoproteins; vRNPs) – is released into the cytoplasm and imported into the nucleus for replication. Subsequently, newly-synthesized viral proteins from the cytoplasm enter the nucleus to form newly-synthesized vRNPs. Later in infection, newly-assembled vRNPs are exported from the nucleus to the cytoplasm to allow for their packaging into progeny virions. The vRNPs contain multiple copies (up to 97) of viral nucleoprotein (NP; 56 kDa) forming a core around which the RNA is helically wrapped (reviewed in [2]). Each NP monomer has at least two nuclear localization sequences (NLS1, spanning residues 1–13 at the N terminus, and NLS2, spanning residues 198–216 in the middle of the protein) that mediate the nuclear import of NP and vRNPs [3-7]. We have previously found that both NLS1 and NLS2 on NP are responsible for mediating the nuclear import of vRNPs purified from influenza A virions in permeabilized cells [7]. We also found that NLS1 of NP is the principal mediator of the nuclear import of incoming vRNPs because NLS1 has higher surface accessibility than NLS2, both within each vRNP molecule and on a greater number of vRNP molecules [8].

Within the nucleus, the original incoming and newly-synthesized negative-sense vRNAs act as templates to transcribe the positive mRNA strand, which is selectively exported into the cytoplasm and used to translate new viral proteins (reviewed in [9]). Some of the newly-synthesized viral proteins (NP; the RNA polymerases PA, PB1, and PB2; the nonstructural protein NS1; the matrix protein M1) are then imported into the nucleus through their respective NLSs. In the nucleus, the newly-synthesized NP, PB1, PB2, PA, and the vRNA assemble into new vRNPs (reviewed in [10]). Subsequently, the newly-assembled vRNPs use the cellular export receptor CRM1 to exit the nucleus through the nuclear pore complexes [11-13].

Nuclear-exported vRNPs are different from incoming vRNPs in that they are somehow prevented from being imported back into the nucleus. It has been demonstrated that association of the vRNPs with the viral protein M1 regulates nuclear trafficking of influenza vRNPs [14,15]. However details of how M1 prevents newly-assembled vRNPs from re-entering the nucleus is unknown. Our hypothesis is that the NLSs on NP are the key determinants for the nuclear transport directionality of the vRNPs by possessing differential exposure. To test this hypothesis, we analyzed the exposure of the NLSs on NP in tissue culture cells infected with influenza A virus. We found that an exposed NLS1 on NP allows newly-synthesized NP to enter the nucleus, but NLS1 becomes masked or hidden once the progeny vRNPs undergo nuclear export. Hidden NLSs on the nuclear-exported vRNPs prevents the nuclear re-entry of the progeny vRNPs. This selective exposure and masking of NLS1 on vRNPs thus constitutes a critical mechanism to regulate the directionality of the nuclear transport of the influenza vRNPs.

## Results

### Specificity of NP antibodies

**Figure 1:**
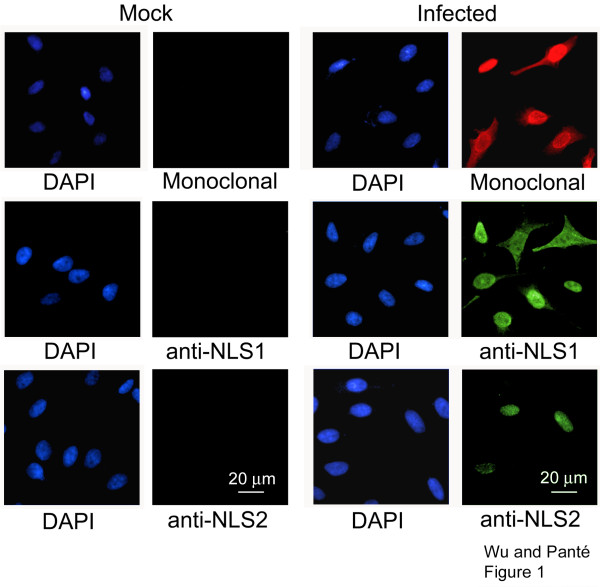
**Specificity of NP antibodies**. Immunofluorescence microscopy of HeLa cells infected with the influenza A virus and immunolabeled with the monoclonal NP antibody, or the polyclonal anti-peptide antibodies that recognize the NLS1 and the NLS2 epitopes of NP. DAPI, a DNA marker, was used to determine the total number of cells present. As a control, a mock infection without influenza A virus was also performed. Cells were fixed and prepared for immunofluorescence microscopy 17 hours after infection.

We have previously generated and characterized two polyclonal anti-peptide antibodies that specifically recognize NLS1 and NLS2 on NP [7,8]. In this study, we used these anti-NLS antibodies to analyze the exposure of these NLSs within cells infected with influenza A virus or transfected with the cDNA of NP. Total NP was detected by using a monoclonal antibody specific for NP. To ensure that all three of the NP monoclonal, anti-NLS1, and anti-NLS2 antibodies were specific for NP and not for components of the cell, we first compared the antibody labeling in infected cells with that in mock-infected cells. We found that each of the respective antibodies gave a strong signal in infected cells compared with mock-infected cells in which no virus was added (Fig. 1). A similar specificity of the anti-NP monoclonal, anti-NLS1, and anti-NLS2 antibodies was observed in cells transfected with the cDNA of NP compared with mock-transfected cells (results not shown).

Besides testing for the specificity of the anti-NP antibodies, the results from Fig. 1 also indicated that NLS1 was generally more exposed than NLS2, and exposed in a greater number of influenza A virus-infected cells. This is in agreement with our previous studies examining the immunogold labeling of purified vRNPs with the anti-NLS1 or anti-NLS2 antibodies [8], and with our conclusion that NLS1 is stronger that NLS2 in mediating the nuclear import of the influenza vRNPs [7].

### Exposure of NLS1 and NLS2 in influenza-infected cells

**Figure 2:**
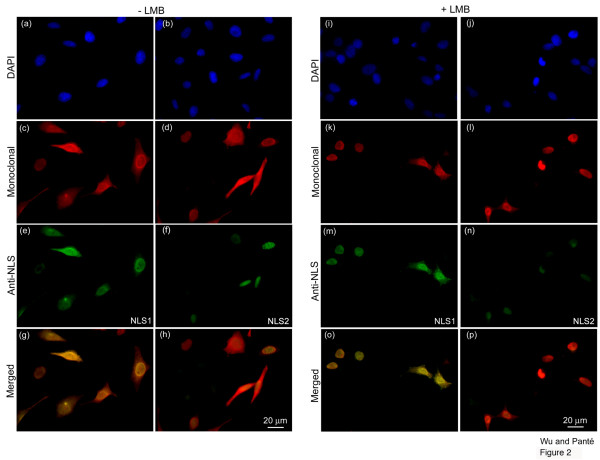
**Exposure of NLS1 and NLS2 in influenza-infected cells**. HeLa cells infected with influenza A virus for 17 hours, in the absence (**a-h**) or presence (**i-p**) of the nuclear export inhibitor LMB, were immunolabeled with DAPI (**a-b **and **i-j**; blue), a monoclonal anti-NP antibody (**c-d **and **k-l**; red), and either the polyclonal anti-NLS1 antibody (**e **and **m**; green) or the polyclonal anti-NLS2 antibody (**f **and **n**; green). Merged images depict merge of the red and green channels for each respective set of cells.

**Figure 3:**
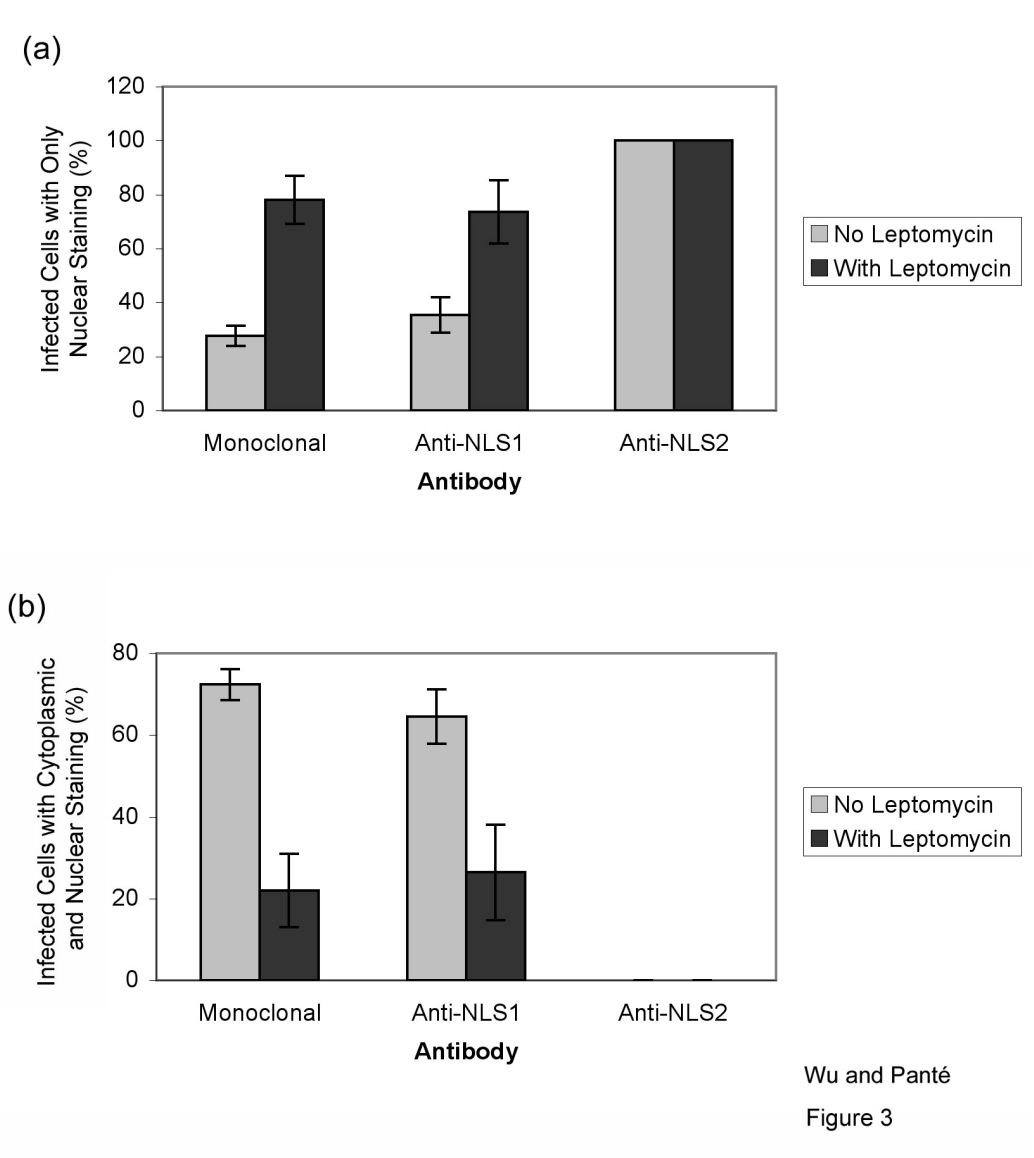
**Quantification of the exposure of NLS1 and NLS2 in influenza-infected cells**. Bar graphs of the percentage of infected cells showing fluorescent staining only in the nucleus (**a**) or both in the cytoplasm and the nucleus (**b**) for the experimental conditions described in Fig. 2. Data shows the mean values and standard error scored from 152 and 82 infected cells in the absence and presence of LMB, respectively.

We performed double-immunolabeling studies with the monoclonal NP antibody in conjunction with either the polyclonal NP anti-NLS1 or with the polyclonal NP anti-NLS2 antibody to analyze the exposure of the NLSs in cells infected with the influenza A virus. As illustrated in Fig. 2, the NP monoclonal antibody detected NP in both the nucleus and cytoplasm of infected cells (Fig. 2c–d), with 28% of the infected cells showing only nuclear staining (Fig. 3a). Similarly, the NLS1 epitope on NP was exposed in both the nucleus and cytoplasm (Fig. 2e). In contrast, the NLS2 epitope was only exposed in the nucleus of the infected cells (Fig. 2f). Quantitative analysis showed that 100% of the infected cells labeled with the anti-NLS2 antibody had only nuclear staining of anti-NLS2, while 35% of the infected cells labeled with the anti-NLS1 antibody had only nuclear staining of anti-NLS1 (Fig. 3a).

**Figure 4:**
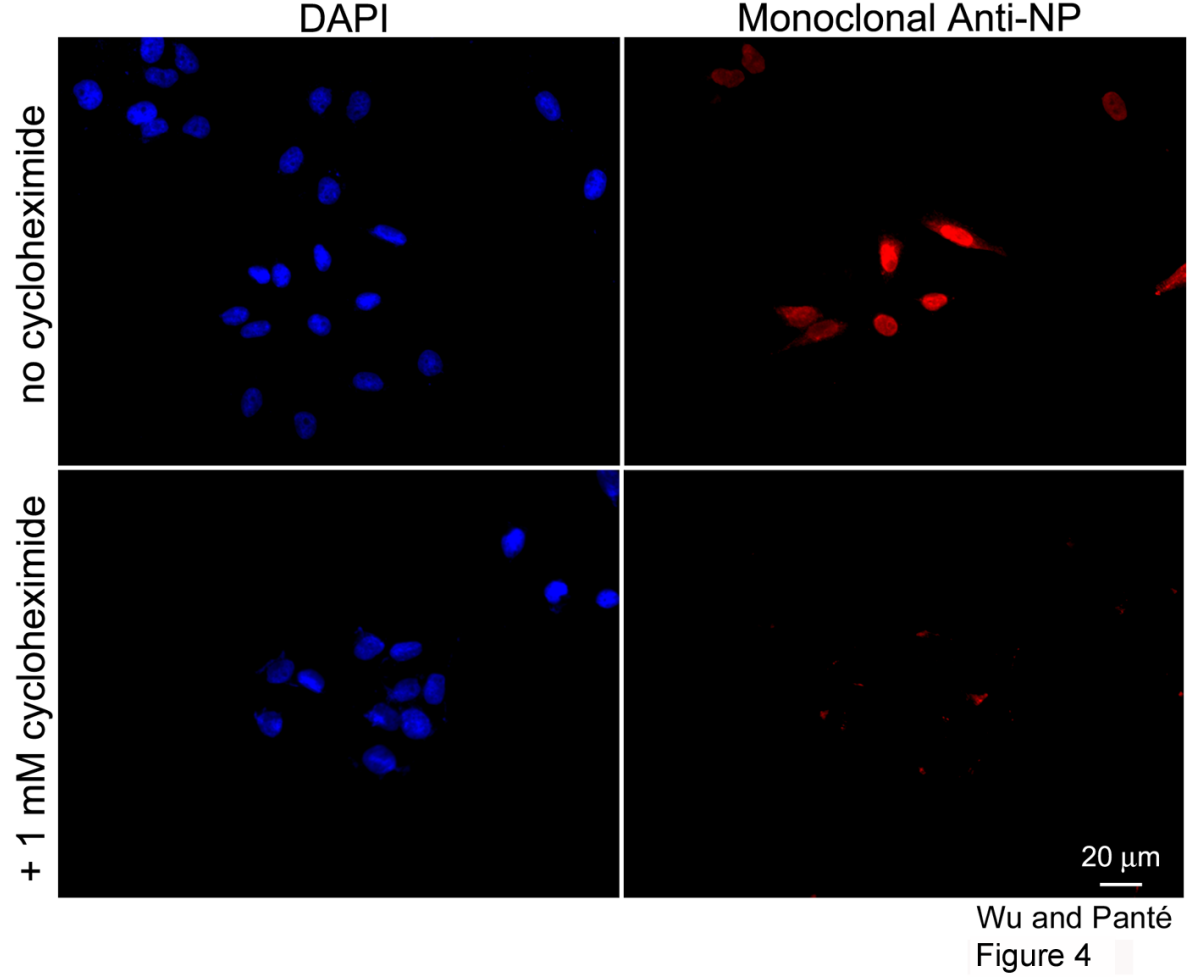
**Localization of newly-synthesized NP in influenza-infected cells**. Immunofluorescence microscopy of HeLa cells infected with the influenza A virus in the absence or presence of the protein synthesis inhibitor, cycloheximide. Cells were fixed and immunolabeled with DAPI and the monoclonal anti-NP antibody 17 hours after infection.

To distinguish between incoming vRNPs and newly synthesized NP and progeny vRNPs, we next performed a similar double-immunolabeling experiment with cells infected with influenza A virus in the presence of cycloheximide (a protein synthesis inhibitor). As illustrated in Fig. 4, there was no NP fluorescence signal in cells treated with cycloheximide. This indicates that the NP being labeled in the infected cells (Fig. 2) represents indeed newly-synthesized NP. Therefore, this limits the type of cytoplasmic NP detected in infected cells to be either newly-synthesized NP or newly-assembled vRNPs that have undergone nuclear export.

From the above results, it was unclear why these infected cells did not contain an exposed NLS2 in the cytoplasm even though the cells contained NP in the cytoplasm. The experiment with cycloheximide helped us to conclude that the cytoplasmic NP does not represent incoming vRNPs. To distinguish whether the cytoplasmic NP is newly-synthesized NP or nuclear-exported vRNPs, we used leptomycin B (LMB) to inhibit the nuclear export of vRNPs. These experiments with LMB detect newly synthesized vRNPs that is trapped in the nucleus. LMB has been successfully used in the past to inhibit the nuclear export of vRNPs in infected cells [11,13]. We repeated these experiments in the presence of LMB, to block vRNP nuclear export and to determine whether the cytoplasmic NP in the infected cells represented newly-synthesized NP or nuclear-exported vRNPs. As documented in Fig. 2k–l, and Fig. 3a, we found that in the presence of LMB 78% of the infected cells showed only nuclear, and no cytoplasmic, NP. Quantitative analysis showed that 22% of the infected cells, however, also still showed cytoplasmic NP in addition to nuclear NP accumulation (Fig. 3b). Because we were inhibiting nuclear export, this cytoplasmic NP represents newly-synthesized NP that had not yet undergone nuclear import.

Consistent with the notion that there were two pools of cytoplasmic NP in infected cells untreated with LMB (newly-synthesized NP and newly-assembled vRNPs that have undergone nuclear export), the experiment in the presence of LMB yielded cells in which the fluorescence intensity of the cytoplasmic NP was less intense than from cells without LMB. Of particular note, this cytoplasmic NP contained an exposed NLS1 (Fig. 2m). In fact, quantitative analysis showed that 26% of infected cells in the presence of LMB still contained both cytoplasmic and nuclear immunostaining with the anti-NLS1 antibody (Fig. 3b). This indicates that newly-synthesized cytoplasmic NP that had not yet undergone nuclear import contains an exposed NLS1 epitope.

A longer time point in infected cells (30 hours instead of 17 hours) was also performed, and there was even less, but still a small amount of cytoplasmic NP staining from both the monoclonal and the anti-NLS1 antibodies (results not shown), indicating that more NP had undergone nuclear import. Taken together, these results indicate that NLS1 (but not NLS2) exposure is a prerequisite for successful nuclear import of newly-synthesized NP.

### Exposure of NLS1 and NLS2 in NP-transfected cells

**Figure 5:**
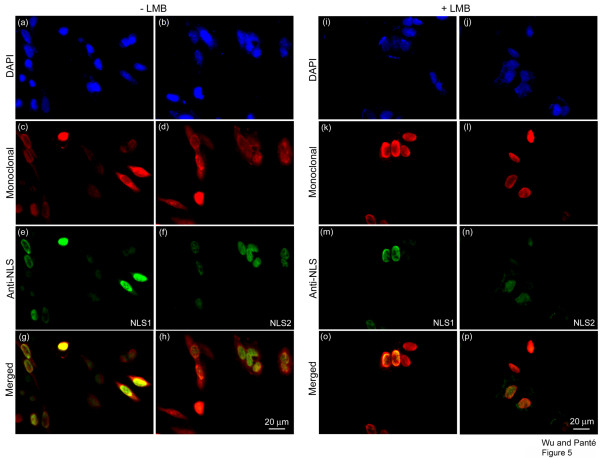
**Exposure of NLS1 and NLS2 in NP-transfected cells**. HeLa cells transfected with the cDNA of NP, in the absence (**a-h**) or presence (**i-p**) of the nuclear export inhibitor LMB, were immunolabeled with DAPI (**a-b **and **i-j**; blue), a monoclonal anti-NP antibody (**c-d **and **k-l**; red), and either the polyclonal anti-NLS1 antibody (**e **and **m**; green) or the polyclonal anti-NLS2 antibody (**f **and **n**; green). Merged images depict merge of the red and green channels for each respective set of cells.

**Figure 6:**
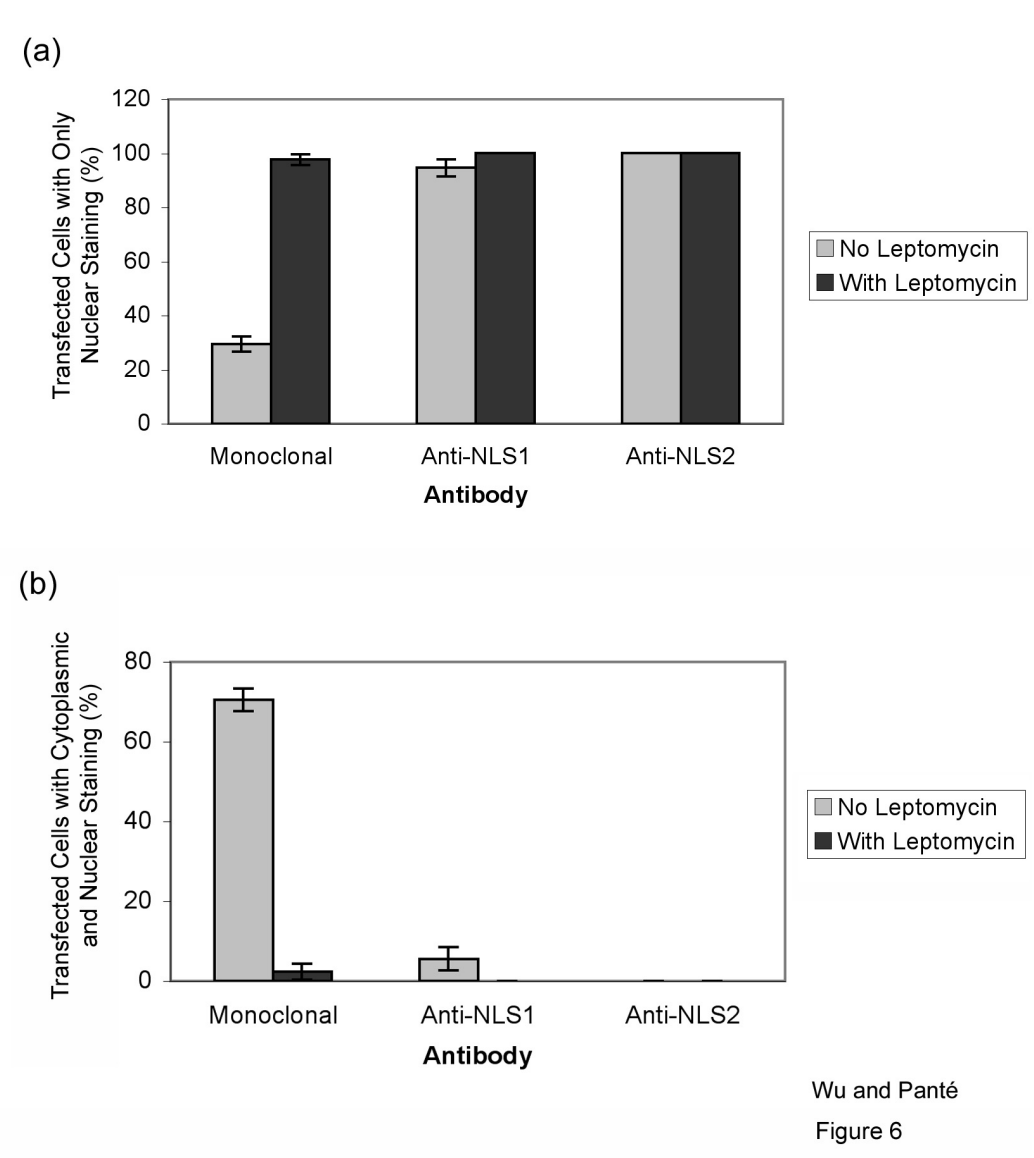
**Quantification of the exposure of NLS1 and NLS2 in NP-transfected cells**. Bar graphs of the percentage of transfected cells showing fluorescent staining only in the nucleus (**a**) or both in the cytoplasm and the nucleus (**b**) for the experimental conditions described in Fig. 5. Data shows the mean values and standard error scored from 288 and 87 transfected cells in the absence and presence of LMB, respectively.

To distinguish any differences in the localization between NP only and NP as part of the vRNP complex, we repeated the immunolocalization experiments in cells transfected with *NP *cDNA. Similar to infected cells, 71% of the transfected cells showed NP in both the cytoplasm and nucleus, as represented by immmunolabeling with the monoclonal anti-NP antibody (Fig. 5c–d, and Fig. 6b). However, NP NLS1 and NLS2 were only exposed in the nucleus, and not cytoplasm, of transfected cells (Fig. 5e–f, and Fig. 6). This contrasted to infected cells, which yielded 65% of the cells with NP NLS1 exposed in the cytoplasm (Fig. 2e and Fig. 3b). According to our results above, this would indicate that the cytoplasmic NP in these transfected cells represented NP that had been nuclear exported, and not newly-synthesized NP, since NLS1 was not exposed in the cytoplasm of transfected cells (Fig. 5e and Fig. 6b) even though 71% of the transfected cells showed NP existing in the cytoplasm (Fig 5c–d, and Fig. 6b). To confirm this and distinguish between the two populations of cytoplasmic NP (nuclear exported or newly-synthesized), we blocked NP nuclear export with LMB. As expected, LMB completely inhibited NP nuclear export, with all the NP being retained in the nucleus of the transfected cells (Fig. 5k–l, and Fig. 6a). This indicates that all the cytoplasmic NP in transfected cells in the absence of LMB (Fig. 5c–d) indeed represented nuclear-exported NP. Since these cytoplasmic NP molecules did not show immunolabeling of NLS1 or NLS2 (Fig. 5e–f), nuclear exported-NP has its NLSs hidden or masked.

### Exposure of NLS1 and NLS2 within the nucleolus

**Figure 7:**
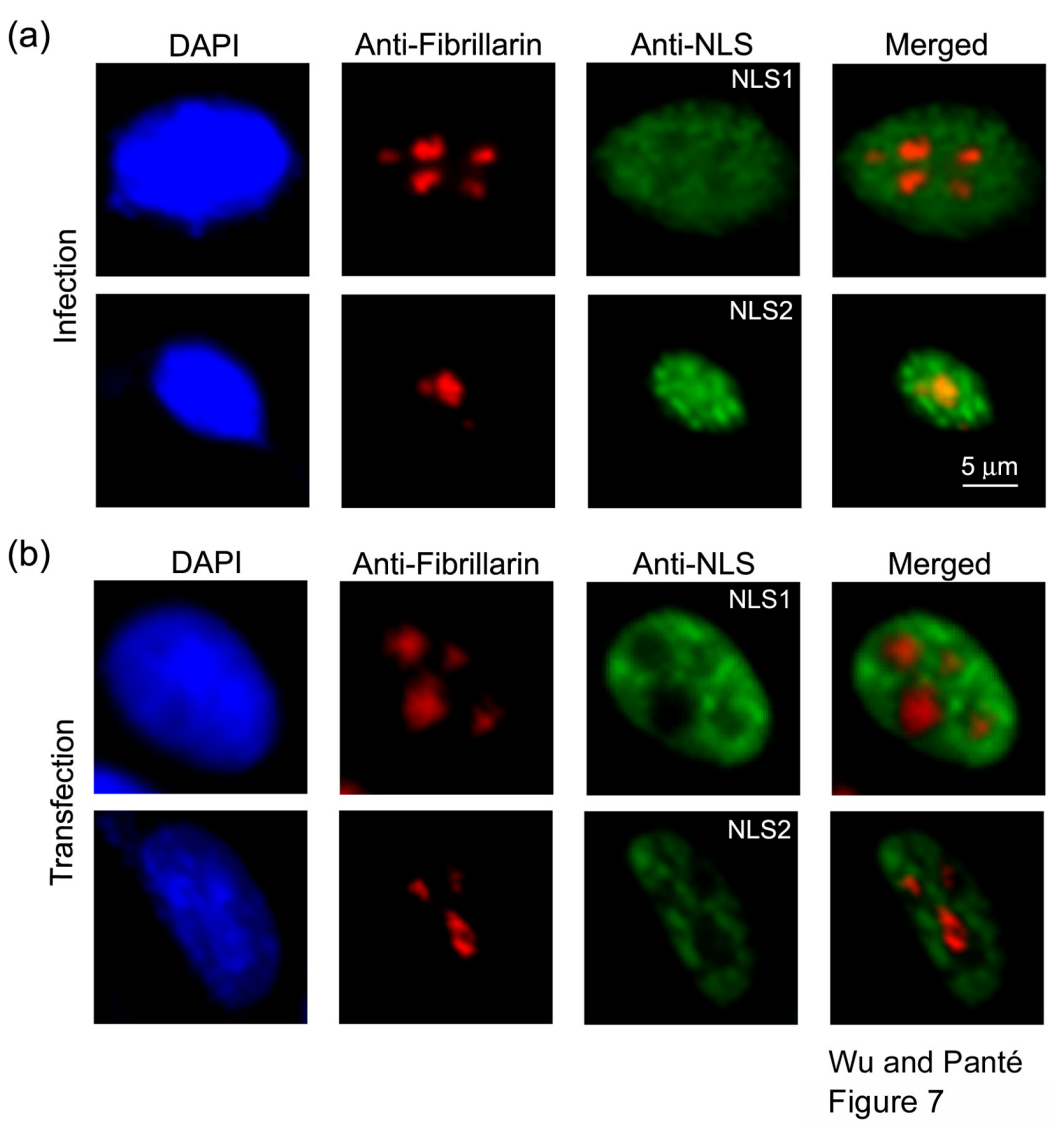
**Exposure of NLS1 and NLS2 within the nucleolus**. Immunofluorescence microscopy of cells infected with the influenza A virus (**a**) or transfected with the cDNA of NP (**b**) and immunolabeled with DAPI, the monoclonal anti-fibrillarin antibody (red), and either the polyclonal anti-NLS1 antibody (green) or the polyclonal anti-NLS2 antibody (green). Merged images of anti-fibrillarin (red) with the corresponding anti-NLS antibody (green) are shown.

We also observed that in infected cells NP localized to distinct nuclear spots, which were reminiscent of nucleoli. To verify this we performed double immunolabeling with the anti-NLS antibodies and a monoclonal antibody against the nucleolar protein fibrillarin. As illustrated in Fig. 7a, we found that in influenza-infected cells, NLS1 was not exposed in the nucleolus. NLS2 was, however, exposed both in the nucleoplasm and the nucleolus. This is in contrast to NP-transfected cells, which have NLS1 and NLS2 exposed in the nucleoplasm, without any exposure in the nucleolus (Fig. 7b). This indicates that one or more components from the influenza virus play a role in allowing NLS2 to become exposed in the nucleolus of influenza A virus-infected cells.

## Discussion

We have previously shown that the NLS1, compared to the NLS2, epitope on NP is more highly exposed throughout each vRNP molecule [8]. This has the consequence that NLS1 is a stronger mediator than NLS2 for nuclear import of vRNPs *in vitro *[7]. In this study, we analyzed the degree of exposure of NLS1 and NLS2 in influenza-infected cells, and found that these NLSs are also differentially exposed in the different cell compartments during the course of an infection. Interestingly, NLS2 was only exposed in the nucleus, while NLS1 was exposed in the cytoplasm and nucleus. By designing experiments that allowed us to detect specific forms of cytoplasmic NP and vRNPs, we found that NLS1 is exposed in newly-synthesized cytoplasmic NP, confirming once more that NLS1, but not NLS2, is especially critical for the nuclear import of influenza NP [6]. The exposure and role of NLS2 in nuclear trafficking of NP and vRNP is less clear. However, our findings that NLS2 is exposed in the nucleolus of infected, but not NP-transfected, cells is in agreement with a role of this sequence for viral replication, as it has been previously demonstrated [16].

**Figure 8:**
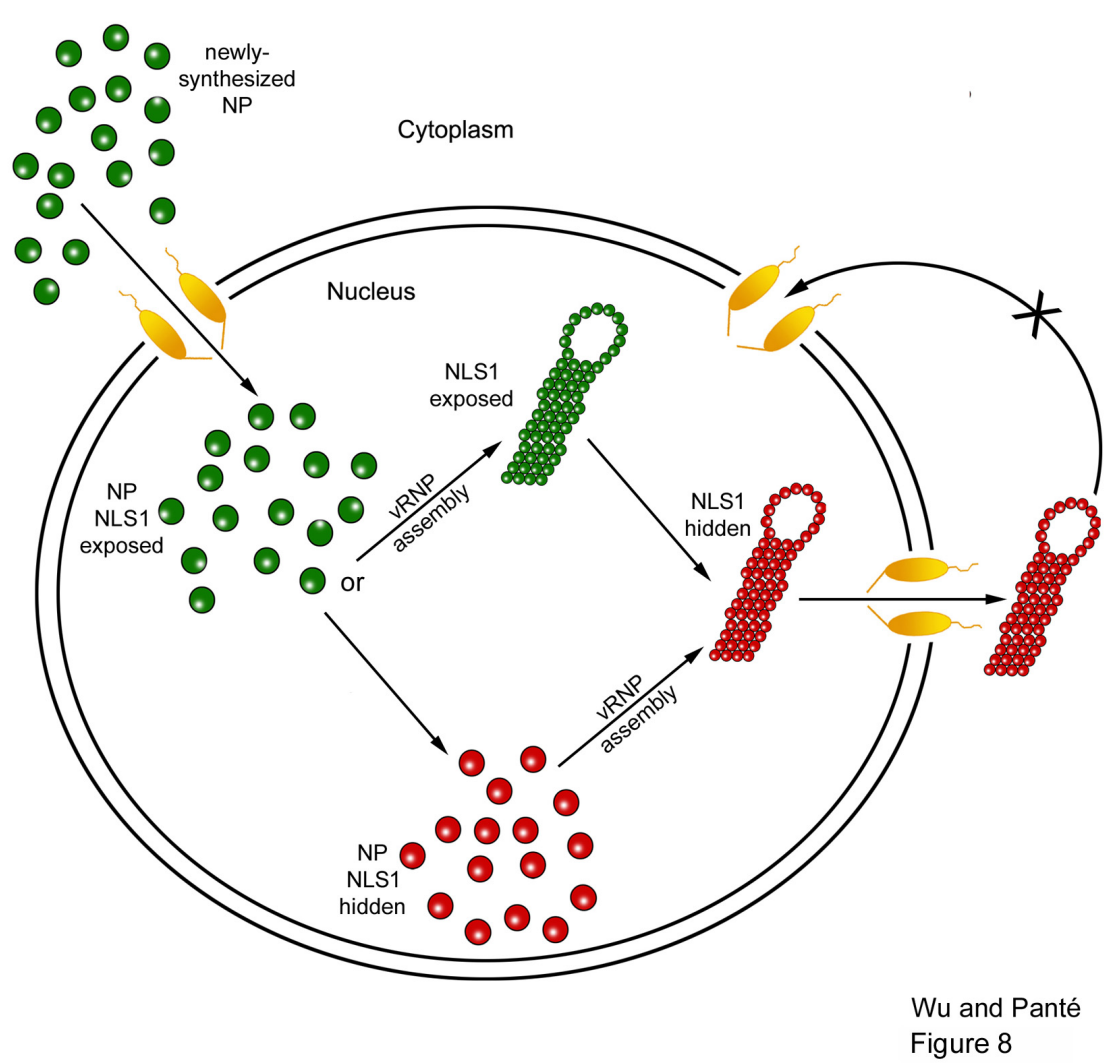
**Model of the exposure of the NLS1 on influenza A NP and its role in cellular nuclear transport**. The exposure and masking of the NP NLS1 mediates the directionality of the nuclear trafficking of influenza NP and vRNP. NP and vRNPs with an exposed NLS1 are represented by green, and NP and vRNPs with a hidden NLS1 are represented by red.

We have also found in this study that nuclear-exported NP contains a masked NLS1, thereby preventing this molecule from re-entering the nucleus. Based on this result, we conclude that the selective exposure and masking of NLS1 constitutes a critical mechanism to regulate the directionality of nuclear trafficking of vRNPs during the influenza A viral life cycle. Our results is consistent with a model (Fig. 8) in which NLS1 is exposed in newly-synthesized NP and also in incoming vRNPs to allow these molecules to bind to cellular importins and enter the nucleus; upon assembly of NP into newly-synthesized vRNPs in the nucleus, NLS1 becomes masked, so after the vRNPs are nuclear exported, they cannot return to the nucleus. The hidden NLS1 epitope thereby critically regulates the directionality of the nuclear transport of newly-assembled vRNPs, driving their uni-directional nuclear export and allowing subsequent cytoplasmic assembly and budding of the complete influenza A virion.

Several putative pathways to encrypt NLS1 on nuclear-exported vRNPs and NP may occur. Since the masking of NLS1 was also observed in transfected, and not only infected, cells, a masked NLS1 epitope is independent of the viral M1 matrix protein, viral RNA, or other influenza A components. NLS1 masking on newly-synthesized vRNP and NP is also unlikely due to NP oligomerization because we have previously demonstrated that NP oligomerized as vRNPs contains an exposed NLS1 [8]. Instead, this NLS masking is likely due to an NP post-translational modification, its binding to a cellular protein, or a conformational change in NP. Which of these mechanisms act to prevent nuclear re-entry awaits further studies.

## Conclusion

Our results indicate that NLS1 is exposed in cells after influenza infection to mediate the nuclear import of incoming vRNPs and newly-synthesized NP. This NLS becomes hidden once progeny vRNPs have been exported from the nucleus. Our data support the model that masking of the NLS1 epitope prevents nuclear re-entry of newly-synthesized vRNPs. The molecular mechanism of this masking awaits further studies, but we believe that this study provides the basic underlying mechanism that regulates the directionality of the nuclear trafficking of influenza vRNPs. We conclude that selective exposure and masking of the NLS1 on the vRNP constitutes a critical mechanism to regulate the directionality of the nuclear transport of vRNPs during the influenza A viral life cycle (Fig. 8).

## Methods

### Cells, viruses, antibodies

HeLa cells (American Type Culture Collection) were cultured in DMEM (HyClone) supplemented with 9% fetal bovine serum (FBS; Sigma) and maintained at 37°C in a humidified atmosphere with 5% CO_2_. Influenza A (A/WSN/1933) *NP *cDNA in the *pCAGGS *vector was kindly provided by Dr. G. Whittaker (Cornell University). The affinity-purified rabbit polyclonal antibodies against the NLSs of NP (NLS1, ^1^MASQGTKRSYEQM^13 ^and NLS2, ^198^KRGINDRNFWRGENGRKTR^216^) were produced by Pacific Immunology, and have been characterized previously [7,8]. The mouse monoclonal NP and fibrillarin antibodies were purchased from Acris and Abcam, respectively. Influenza A virus (A/Aichi/1968) was obtained from Charles River Laboratories.

### Influenza infection

HeLa cells were plated at 30% confluency the day before infection in growth media containing 9% FBS onto 12-mm glass cover slips in 12-well plates. The next day, the cells were washed with phosphate buffered saline (PBS), and then 1 ml of growth media containing 0.2% FBS was applied to each well. 30 μl of the influenza A virus at 2 mg/ml (MOI of 1) were applied to the cells. The virus was allowed to adsorb to the surface of the cells for 40 minutes at room temperature, with gentle rocking every 10–15 minutes. The media containing the virus was then removed, and replaced with 1 ml of media containing 2% FBS. The cells were incubated for 17 or 30 hours in a 37°C incubator containing 5% CO_2_. After these incubation times, the cells were prepared for immunofluorescence microscopy as described below.

For some experiments, the protein synthesis inhibitor cycloheximide (Sigma, St. Louis) at a final concentration of 1 mM was added to the 2% FBS medium. To inhibit nuclear export, leptomycin B (LMB; Sigma) was added to the cells 6 hours after replacing the media containing 2% FBS, and cells were incubated for a total of 17 or 30 hours at 37°C. LMB was used at a concentration of 11 nM, which is effective for the inhibition of the nuclear export of NP and vRNPs, as previously reported [6,11].

### Transfections

Transfection of HeLa cells with *NP-pCAGGS *was carried out on glass coverslips with Lipofectamine 2000 (Invitrogen) according to the manufacturer's protocol. For some experiments, cycloheximide (final concentration 1 mM) was added after media was changed, and the cells were incubated for 24 hours at 37°C. To inhibit nuclear export, LMB (final concentration 11 nM) was added 6 hours after the change in media, and the cells were incubated for a total of 30 hours at 37°C. After these incubation times, the cells were prepared for immunofluorescence microscopy as described below.

### Immunofluorescence microscopy

After infection or transfection, cells were washed three times with PBS, fixed for 10 minutes with 4% paraformaldehyde, permeabilized with 0.2% Triton X-100 (Sigma) in PBS containing 10% goat serum (Sigma), and labeled with the NP or the fibrillarin monoclonal antibody and either the NP polyclonal anti-NLS1 or the NP polyclonal anti-NLS2 antibody for 1 hour, followed by incubation with secondary antibodies (goat anti-mouse rhodamine and goat anti-rabbit fluorescein, both from Invitrogen). After washing, coverslips were mounted with Prolong Gold antifade containing DAPI (Invitrogen). Fluorescence microscopy was performed on a Zeiss Axioplan 2.

## Competing interests

The authors declare that they have no competing interests.

## Authors' contributions

WWHW carried out the experiments and drafted the manuscript. NP conceived the study and experimental design, coordinated the study, and helped to draft the manuscript. All authors read and approved the final manuscript.
